# A Dual-Band Wide-Input-Range Adaptive CMOS RF–DC Converter for Ambient RF Energy Harvesting

**DOI:** 10.3390/s21227483

**Published:** 2021-11-10

**Authors:** Bo-Ram Heo, Ickjin Kwon

**Affiliations:** Department of Electrical and Computer Engineering, College of Information Technology, Ajou University, Suwon 16499, Korea; ramob94@ajou.ac.kr

**Keywords:** adaptive, CMOS rectifier, dual-band, RF–DC power converter, RF energy harvesting, wide input range

## Abstract

In this paper, a dual-band wide-input-range adaptive radio frequency-to-direct current (RF–DC) converter operating in the 0.9 GHz and 2.4 GHz bands is proposed for ambient RF energy harvesting. The proposed dual-band RF–DC converter adopts a dual-band impedance-matching network to harvest RF energy from multiple frequency bands. To solve the problem consisting in the great degradation of the power conversion efficiency (PCE) of a multi-band rectifier according to the RF input power range because the available RF input power range is different according to the frequency band, the proposed dual-band RF rectifier adopts an adaptive configuration that changes the operation mode so that the number of stages is optimized. Since the optimum peak PCE can be obtained according to the RF input power, the PCE can be increased over a wide RF input power range of multiple bands. When dual-band RF input powers of 0.9 GHz and 2.4 GHz were applied, a peak PCE of 67.1% at an input power of −12 dBm and a peak PCE of 62.9% at an input power of −19 dBm were achieved. The input sensitivity to obtain an output voltage of 1 V was −17 dBm, and the RF input power range with a PCE greater than 20% was 21 dB. The proposed design achieved the highest peak PCE and the widest RF input power range compared with previously reported CMOS multi-band rectifiers.

## 1. Introduction

Energy harvesting technology is one of the core technologies required for the Internet of Things (IoT) because it can drive wireless sensors without replacing or charging the battery. Radio frequency (RF) energy harvesting, which receives ambient RF signals and converts them into DC power, is suitable as a power supply for IoT operation, because available energy can be obtained regardless of external environment or temperature fluctuations.

In an RF energy-harvesting system, an RF–DC converter harvests RF energy from ambient RF sources to obtain an output DC voltage for the power supply of IoT sensors/devices [1,2,3,4,5,6,7,8,9]. However, since the ambient RF energy available in a single band is very small, it is not sufficient to provide the power needed to operate the wireless sensor [10]. To increase the power that can be obtained by RF energy harvesting, research on RF rectifiers operating in multi-bands have been reported in recent years [11,12,13,14]. The dual-band RF–DC converter described in [12] integrates matching networks and band-pass and band-stop filters using a system-in-package (SiP) technique for dual-band operation in a compact solution. The dual-band RF–DC converter presented in [13] uses the internal threshold voltage cancellation technique to reduce the threshold voltage of the forward-biased transistors and increases the threshold voltage of the reverse-biased transistors to maintain a high power conversion efficiency (PCE). However, the reported multi-band rectifiers have relatively low efficiency compared to single-band rectifiers [11,12], and the peak PCE can be obtained only at a specific input power, so the RF input power range in which high PCE can be obtained is narrow [13,14]. 

In actual application, the distance from the RF source to the RF energy harvester is very diverse, and the input power of the rectifier also varies greatly depending on the difference in distance. Particularly, in a multi-band rectifier, it is very important to maintain high power conversion efficiency in a wide input power range because the RF source for each band is different, and the variation of the input RF power range is large. The single-band rectifier reported in [5] extends the input power range by a dual-path configuration with a low-threshold voltage path and a high-threshold voltage path. However, such a dual-path rectifier is difficult to apply to a general CMOS technology in which the types of MOSFETs having different threshold voltages are limited, and the RF input power range with high PCE is significantly limited because the threshold voltage difference of the available MOSFETs is limited. In addition, in the low-threshold voltage path, the PCE degrades because the leakage current increases significantly with the increase of the RF input power.

In this paper, a dual-band adaptive CMOS RF–DC converter operating in the 0.9 GHz and 2.4 GHz bands is proposed for ambient RF energy harvesting. Compared with dedicated RF energy sources, ambient RF energy is available from sources such as cellular base stations, Wi-Fi transmitters, satellite communication transceivers, and TV transmitters [15]. The proposed RF–DC converter is applied to multi-band ambient RF energy harvesting that simultaneously harvests multi-band RF energy available in GSM (900 MHz), ISM (902–928 MHz), and Wi-Fi (2450 MHz) bands. As more RF energy can be harvested, the available energy in ambient RF energy harvesting applications is increased. Since the proposed multi-band rectifier adopts a multi-band input impedance-matching network, it is possible to harvest RF energy from several frequency bands, thus lowering the minimum RF power required in each frequency band to obtain the required output DC voltage. 

In order to solve the problem that the PCE of the multi-band rectifier is greatly degraded according to the RF input power because the available RF input power range is different according to the frequency band, the proposed multi-band rectifier adopts an adaptive rectifier configuration that changes the operation mode so that the number of stages is optimized. Since the optimum peak PCE can be obtained according to the RF input power, the PCE can be increased over a wide RF input power range. The proposed adaptive RF–DC converter is easily applied to the existing CMOS technology because it does not require MOSFETs with different threshold voltages. High PCE is maintained at high RF input power because the power conversion efficiency is not significantly degraded due to the increased leakage current through the MOSFETs with a low threshold voltage. In addition, the RF input power range can be significantly extended by adaptively configuring the number of stages.

## 2. Proposed Dual-Band RF–DC Converter

### 2.1. Adaptive RF–DC Converter Design

In a multi-band RF–DC converter consisting of separate single-band input impedance-matching network and rectifier for each band, only the RF power available in each frequency band is applied to the rectifier, and the power conversion efficiency decreases at low RF input power [15,16]. In addition, an output DC combiner circuit using active elements is required to add the output voltage of each rectifier, resulting in additional power loss and lower power conversion efficiency. On the other hand, in a multi-band RF–DC converter composed of a multi-band input impedance-matching network and a single rectifier for multi-band, the ambient RF power of each band is applied to the same rectifier. Accordingly, the total RF input power applied to the rectifier increases, thereby increasing the output DC voltage. In addition, there is no further reduction in power conversion efficiency because a DC combiner is not required [17,18,19].

The multi-band RF–DC converter increases the available RF input power to obtain an output DC voltage because it collects ambient RF energy in multiple frequency bands. However, since the available RF input power varies according to the frequency band, and the rectifier generally has a peak PCE at a specific RF input power, the PCE of the multi-band rectifier falls significantly depending on the RF input power range. 

To solve this problem, the proposed multi-band RF–DC converter adopts an adaptive rectifier configuration that changes the operation mode so that the number of stages is optimized. The proposed design achieves high PCE over a wide RF input power range because the operating mode is adaptively changed according to the RF input power to have the optimal peak PCE at different RF input powers. The proposed dual-band RF–DC converter adopts an adaptive rectifier having a dual-band input impedance-matching circuit of 0.9 GHz and 2.4 GHz and a control circuit that controls the adaptive operation according to the RF input power.

**Figure 1:**
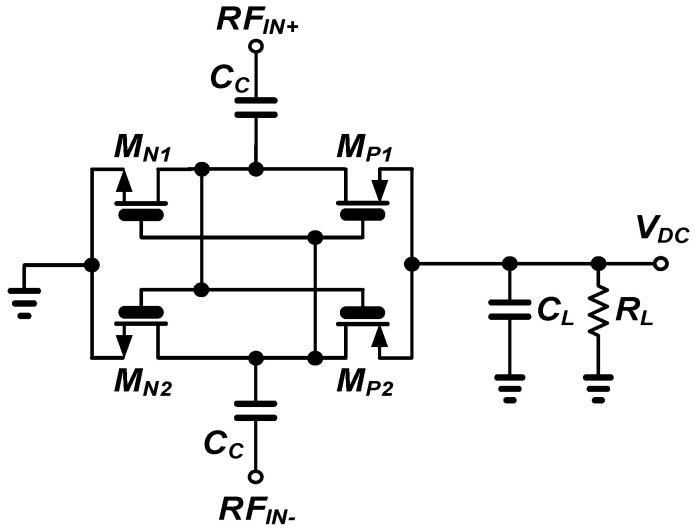
Differential cross-coupled rectifier unit with thick-oxide MOSFET switches.

Figure 1 shows the cross-coupled differential rectifier unit based on thick-oxide MOSFET as the rectifying device. The cross-coupled rectifier with thick oxide MOSFETs achieves higher PCE than conventional rectifiers because it reduces the reverse leakage current and thus reduces the RF input power required to obtain the same output voltage [20].

**Figure 2:**
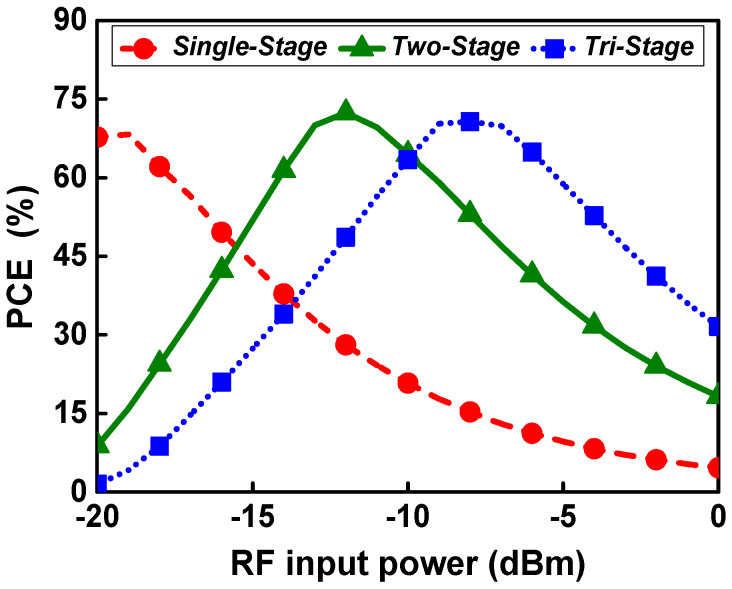
PCE according to the RF input power of rectifiers with a different number of stages.

Figure 2 shows the power conversion efficiency according to the RF input power when composed of a different number of stages based on the unit rectifier. The power conversion efficiency (PCE) is expressed as follows:

(1) $$\mathrm{PCE}(\%)=\frac{V_{DC}{}^{2}}{R_{L}\times P_{IN}}\times 100$$

where *V_DC_* is the output DC voltage, *R_L_* is the load resistance, and *P_IN_* is the RF input power. RF input signals of 0.9 GHz and 2.4 GHz are simultaneously applied to the rectifier with the same input power. Therefore, the RF input power of each band is 3 dB lower than the total RF input power.

As shown in Figure 2, a single-stage converter has a higher PCE at an RF input power below −16 dBm, and a two-stage converter has higher PCE over an input power range from −13 dBm to −10 dBm. The single-stage converter achieves a peak PCE of 67.7% at −19 dBm input, and the two-stage converter achieves a peak PCE of 72.4% at −13 dBm input power. The RF input power at which peak PCE is achieved depends on the number of stages, with fewer stages achieving peak PCE at lower RF input power. Therefore, the proposed circuit adopts an adaptive RF–DC converter whose number of stages varies according to the input power in order to obtain high PCE over a wider input power range. The proposed adaptive RF–DC converter operates in a single-stage configuration at an input power of less than −16 dBm and in a two-stage configuration at an input power of −16 dBm or more to obtain the optimal PCE according to the RF input power. Thus, the RF input power range with high efficiency is improved. 

**Figure 3:**
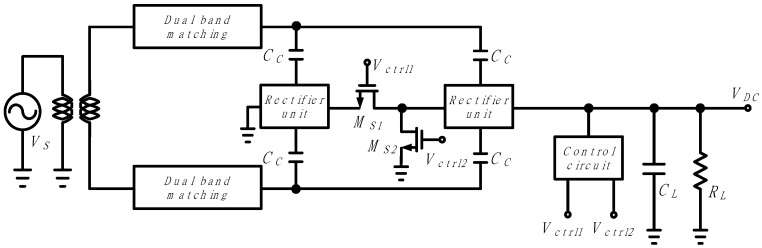
Block diagram of the proposed dual-band adaptive RF–DC converter.

Figure 3 shows the circuit diagram of the proposed dual-band adaptive RF–DC converter. The RF–DC converter operates an adaptive rectifier based on two unit rectifiers. It consists of two switches (*M*_*S*1_, *M*_S2_) that change the configuration to either a single-stage rectifier or a series two-stage rectifier, and a control circuit that senses the output voltage and generates control voltages (*V*_*ctrl*1_, *V*_*ctrl*2_) according to the input power. Therefore, the proposed RF–DC converter operates adaptively in a single-stage configuration or a series two-stage configuration depending on the RF input power based on two unit rectifiers. The dual-band (0.9 GHz, 2.4 GHz) RF signals are applied to the input impedance-matched rectifier through the multi-band impedance matching network. The rectified DC voltage *V_DC_* is output to a load composed of the load capacitor *C_L_* and the load resistor *R_L_*.

**Figure 4:**
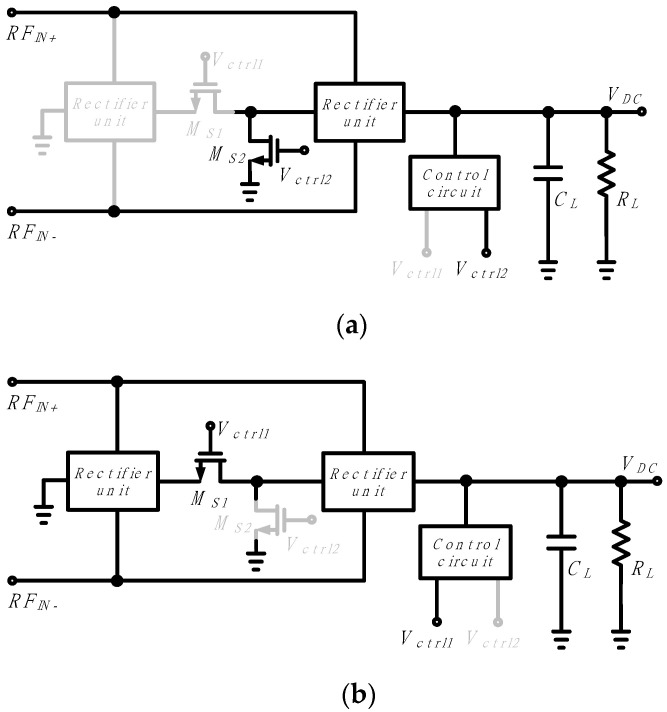
Operation of an adaptive RF–DC converter according to the input power: (**a**) Single-stage configuration at low input; (**b**) Series two-stage configuration at high input.

Figure 4 shows the two adaptive operation configurations of the proposed adaptive CMOS RF–DC converter. At low input power of less than −16 dBm, the control voltages *V*_*ctrl*1_ and *V*_*ctrl*2_ become low and high, respectively, *M*_*S*1_ is turned off, and *M*_*S*2_ is turned on, so the rectifier circuit operates in a single-stage configuration, as shown in Figure 4a. On the other hand, at high input power of −16 dBm or higher, *V*_*ctrl*1_ and *V*_*ctrl*2_ become high and low, respectively, *M*_*S*1_ turns on, and *M*_*S*2_ turns off, so the rectifier circuit operates in a series two-stage configuration as shown in Figure 4b. 

### 2.2. Adaptive Mode Control Circuit Design

**Figure 5:**
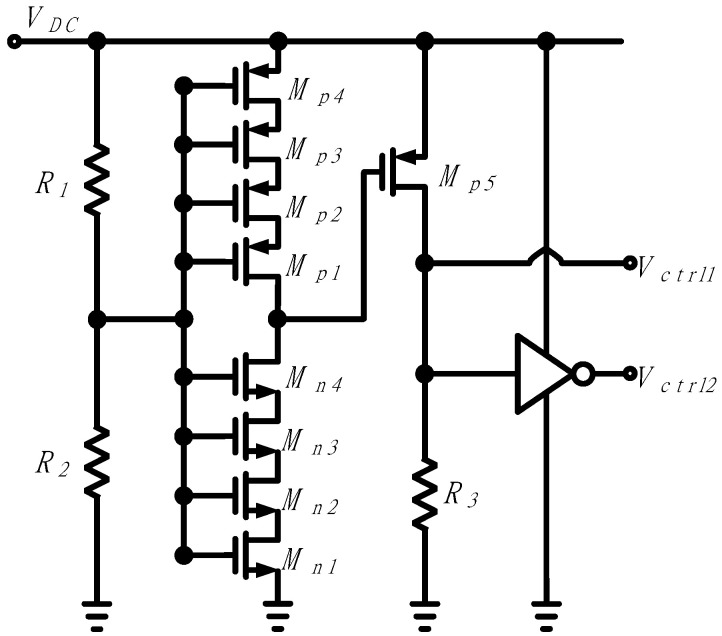
Control circuit for generating control voltages that switch adaptive operation.

Figure 5 shows the circuit diagram of the control circuit that determines the adaptive mode of operation. The control circuit senses the output DC voltage *V_DC_* of the rectifier and generates two control voltages *V*_*ctrl*1_ and *V*_*ctrl*2_ for MOSFET switch control. When the voltage *V_DC_* is at a low level, *M*_*p*1_ to *M*_p4_ are on, *M*_*n*1_ to *M*_n4_ are off, and *M*_*p*5_ is off, and the control voltages *V*_*ctrl*1_ and *V*_*ctrl*2_ are low and high, respectively. On the other hand, when *V_DC_* is at a high level, *M*_*p*1_ to *M*_*p*4_ are off, *M*_*n*1_ to *M*_*n*4_ are on, and *M*_*p*5_ is on, so *V*_*ctrl*1_ and *V*_*ctrl*2_ are high and low, respectively. 

**Figure 6:**
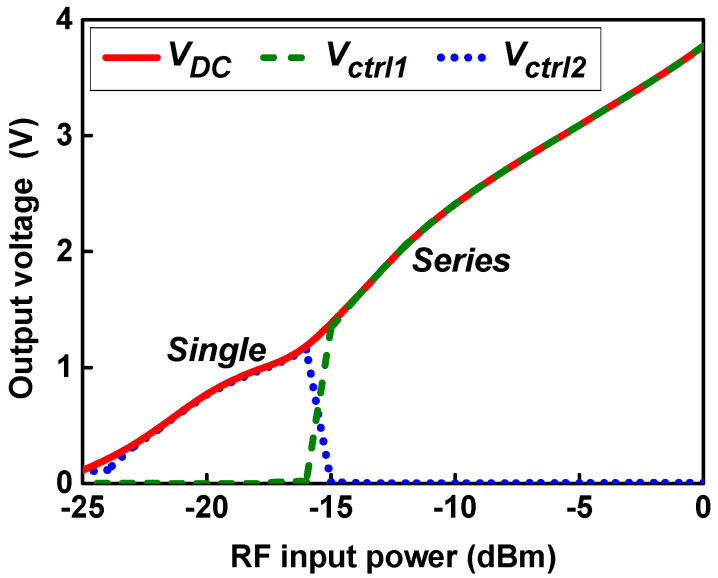
Output voltage and control voltages according to the RF input power.

Figure 6 shows the output voltages *V_DC_*, *V*_*ctrl*1_, and *V*_*ctrl*2_ of the control circuit according to the RF input power. The input power for switching for adaptive operation is designed to be −16 dBm. *V*_*ctrl*1_ and *V*_*ctrl*2_ voltages are switched according to the magnitude of the *V_DC_* voltage depending on the RF input power and operate in a single-stage configuration at input power below −16 dBm and in a series two-stage configuration at input power above −16 dBm. 

**Figure 7:**
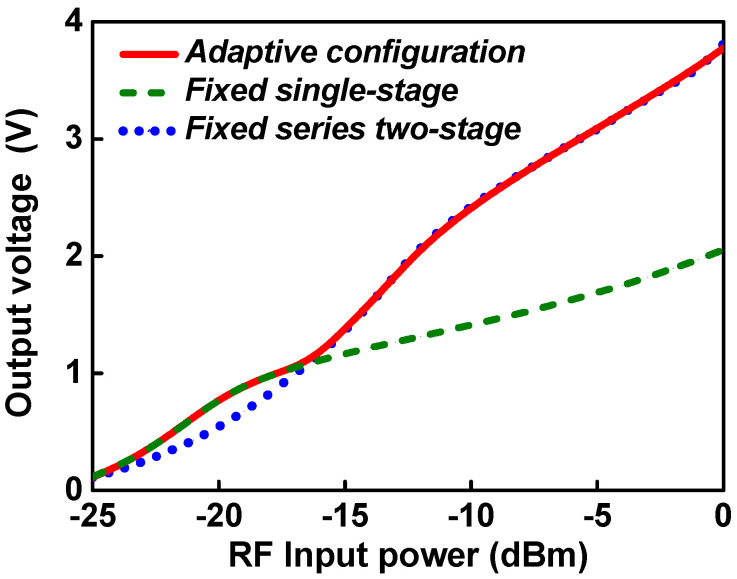
Output voltage according to RF input power for each rectifier configuration.

Figure 7 shows the output voltage according to the RF input power of the proposed RF–DC converter for fixed single-stage configuration, fixed series two-stage configuration, and adaptive configuration. As the RF input power increases, the DC output voltage of each configuration increases. The DC output voltage of the single-stage configuration is higher at low RF input power, but the DC output voltage of the series two-stage configuration is higher at RF input power above −16 dBm. The proposed adaptive RF–DC converter operates in a single-stage configuration at input powers lower than −16 dBm and in a series two-stage configuration at input powers higher than −16 dBm. Thus, over the operating RF input range, the adaptive RF–DC converter achieves a higher DC output voltage than the fixed configuration. 

**Figure 8:**
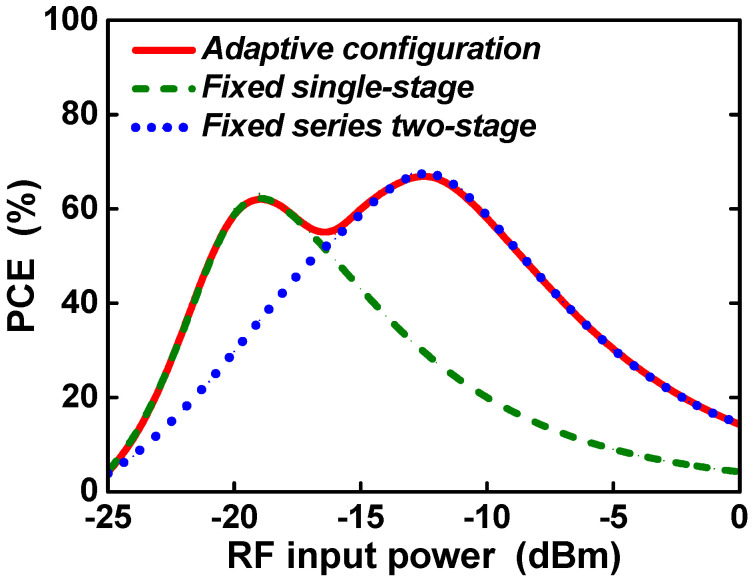
PCE according to RF input power for each rectifier configuration.

Figure 8 shows the PCE according to the RF input power for each rectifier configuration. The single-stage configuration achieves a peak PCE of 62.9% at an RF input power of −19 dBm, and a series two-stage configuration achieves a peak PCE of 67% at an RF input power of −13 dBm. On the other hand, the proposed adaptive RF–DC converter achieves peak PCE of 62.9% and 67%, respectively, at both −19 dBm and −13 dBm RF input power, and the PCE is greatly improved over the wide RF input power range between the two input powers. In the proposed adaptive RF–DC converter, the input power range to achieve efficiency above 80% of the peak PCE is 11 dB, and the input power range is significantly improved by more than 6 dB compared with the fixed single-stage configuration.

### 2.3. Dual-Band Impedance-Matching Circuit Design

**Figure 9:**
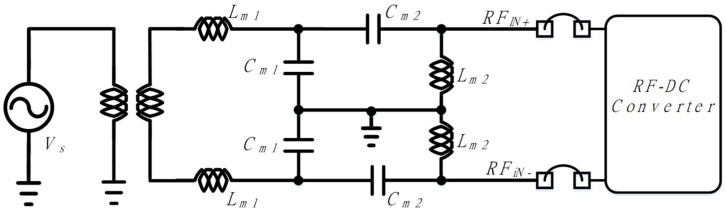
Schematic of the dual-band impedance-matching network.

Figure 9 shows the schematic of a dual-band input impedance-matching network. An off-chip balun is used for the differential RF input, and the input impedance-matching network consists of off-chip inductors and capacitors. *L*_*m*2_ and *C*_*m*2_ are designed for low-band 0.9 GHz input impedance matching, and *L*_*m*1_ and *C*_*m*2_ are designed for high-band 2.4 GHz input impedance matching. 

**Figure 10:**
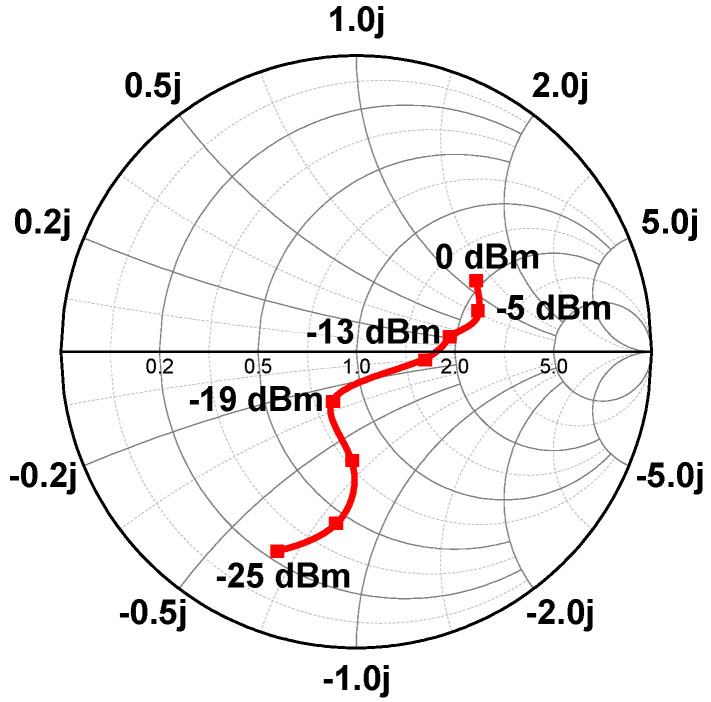
Input impedance of the RF–DC converter according to the RF input power.

Figure 10 shows the input impedance of the RF–DC converter when the RF input power varies from −25 dBm to 0 dBm. Since the input impedance of the RF–DC converter varies considerably with the RF input power, the use of a fixed input impedance-matching network circuit results in impedance matching only at a specific RF input power. Therefore, in order to optimize the power conversion efficiency over the wide input power range of the RF–DC converter, the impedance-matching circuit of the RF–DC converter needs to be optimized to achieve input impedance matching at optimal RF input power.

In the proposed adaptive RF–DC converter, it operates in a single-stage configuration or a series two-stage configuration depending on the RF input power range, obtaining peak PCE at input powers of −19 dBm and −13 dBm, respectively. For optimal input impedance-matching circuit design, each input impedance-matching circuit is designed under different RF input power conditions, and the power conversion efficiency of each rectifier is compared. 

**Figure 11:**
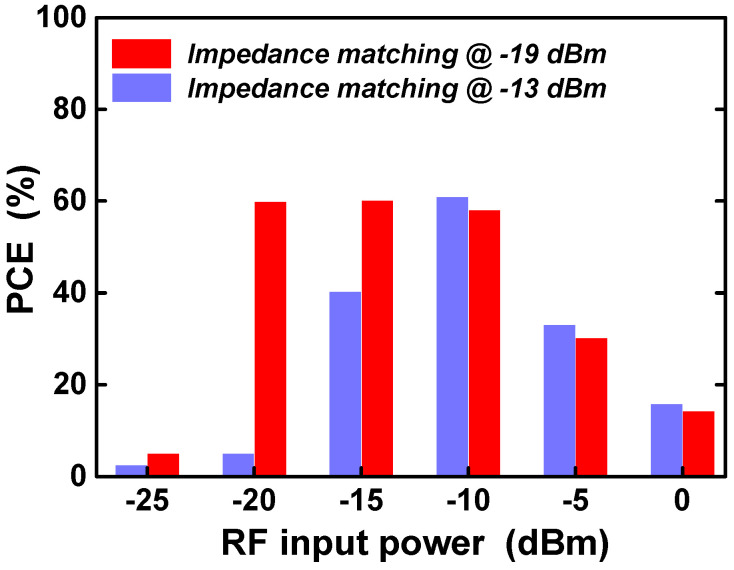
PCE according to RF input power under different input impedance matching values.

Figure 11 shows the PCE of the RF–DC converter according to the RF input power when the input impedance-matching circuit of the RF–DC converter is optimized for different RF input power conditions. As shown in Figure 11, the design with the impedance-matching circuit optimized at the −19 dBm input power condition shows higher power conversion efficiency over a wider input power range than the design with the impedance-matching circuit optimized at the −13 dBm input power condition. In the low input power range below −15 dBm, the PCE difference between the two designs increases significantly. In general, the average power of the ambient RF carrier obtained by RF energy harvesting is less than −10 dBm [20]. Therefore, in this design, the input impedance-matching circuit is optimized for the input power condition of −19 dBm to obtain a high power conversion efficiency over the wide available RF input power range.

## 3. Results and Discussion

**Figure 12:**
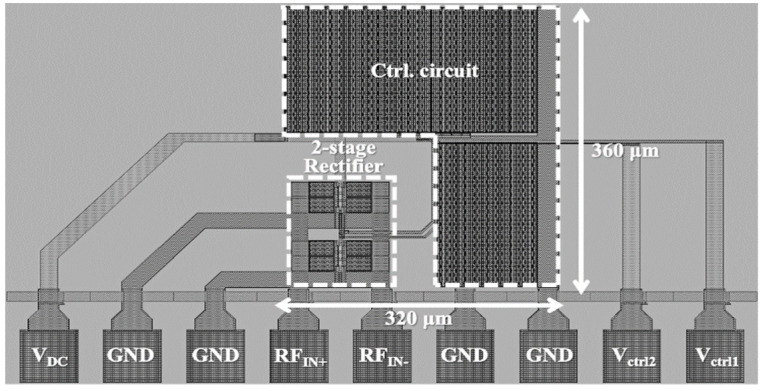
Chip layout of the proposed CMOS RF–DC converter.

The proposed dual-band adaptive RF–DC converter is designed using a 0.18 μm RF CMOS technology provided by MagnaChip foundry. Figure 12 shows the chip layout of the proposed dual-band adaptive RF–DC converter. The chip area of the RF–DC converter composed of two differential cross-coupled rectifier units, MOSFET switches, and control circuit is 320 μm × 360 μm. A Cadence Spectre RF simulator was used in the post-layout simulations. Post-layout simulations of the entire circuit including the off-chip impedance-matching network were performed. The RF models of the off-chip discrete components were provided by Murata. 

**Figure 13:**
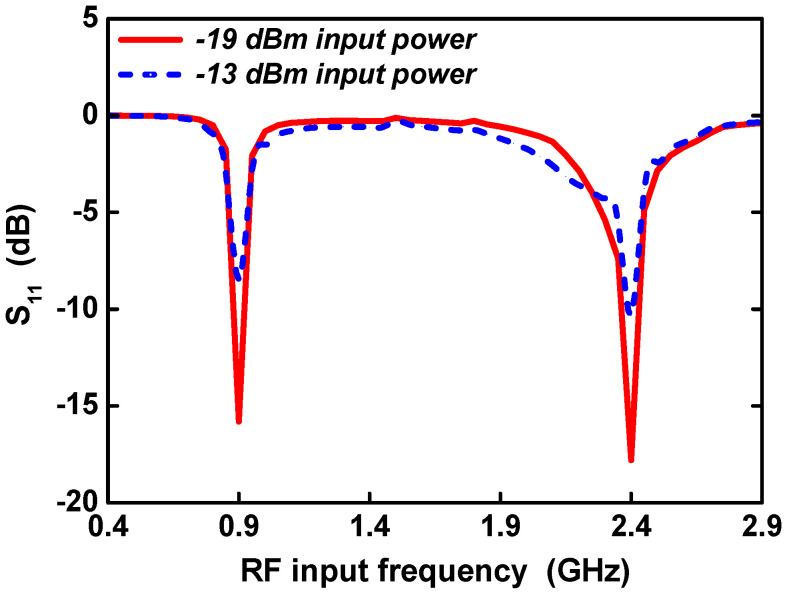
Reflection coefficient (*S*_11_) versus RF input frequency at different input powers.

Figure 13 shows the post-layout simulated reflection coefficient *S*_11_ according to the RF frequency for different RF input powers of −19 dBm and −13 dBm. When the RF input power was −19 dBm, the simulated *S*_11_ was −15.8 dB at 0.9 GHz and −17.8 dB at 2.4 GHz. When the RF input power was −13 dBm, the simulated *S*_11_ was −8.5 dB at 0.9 GHz and −10.3 dB at 2.4 GHz.

**Figure 14:**
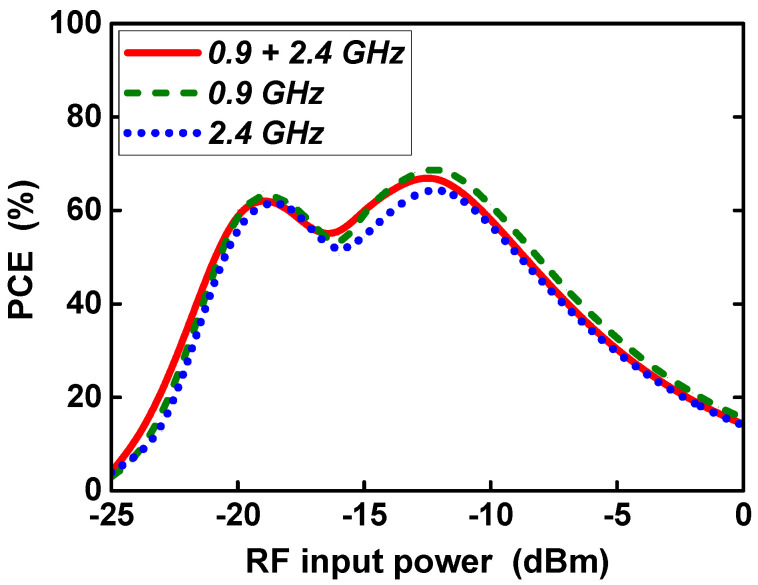
PCE according to RF input power at dual-band and single-band input.

Figure 14 shows the post-layout simulated power conversion efficiency according to RF input power for single-band operation and dual-band operation. In single-band operation, the RF input signal was applied only in the 0.9 GHz or 2.4 GHz band, and the RF input power of each band was equal to the total RF input power. In dual-band operation, since 0.9 GHz and 2.4 GHz RF input signals were simultaneously applied with the same input power, the RF input power of each band was 3 dB lower than the total RF input power. When applying a single-band RF input power of 0.9 GHz, a peak PCE of 69.3% at an input power of −12 dBm and a peak PCE of 64% at an input power of −19 dBm were achieved. When applying a single-band RF input power of 2.4 GHz, a peak PCE of 64% at an input power of −12 dBm and a peak PCE of 61.9% at an input power of −19 dBm were achieved. When applying dual-band RF input power of 0.9 GHz and 2.4 GHz, a peak PCE of 67.1% at an input power of −12 dBm and a peak PCE of 62.9% at an input power of −19 dBm were achieved. The input power that could achieve a PCE greater than 20% of the peak PCE ranged from −23 dBm to −2 dBm, resulting in an RF input power range of 21 dB.

Table 1 shows the simulated *S*_11_, output voltage, and peak PCE results at different temperatures. A maximum PCE of 63.6% was achieved even when the temperature was significantly increased up to 70 °C, demonstrating that it maintained high efficiency against environmental temperature variations.

Table 2 shows the performance summary of the proposed circuit and a comparison with the previously reported works. The proposed dual-band adaptive RF–DC converter achieved 67.1% peak PCE in dual-band operation of 0.9 GHz and 2.4 GHz, enabling efficient multi-band RF energy harvesting. The RF input sensitivity to obtain an output DC voltage of 1 V was −17 dBm, and the RF input power range was 21 dB to maintain a PCE of more than 20%. Compared with previous CMOS multi-band rectifiers, higher peak PCE and wider RF input power range were achieved. Therefore, efficient ambient RF energy harvesting is possible in a wide RF input power range of multi-bands in which the RF input power is significantly different according to frequency bands. 

## 4. Conclusions

In this paper, a dual-band adaptive CMOS RF–DC converter for ambient RF energy harvesting is proposed. The proposed dual-band RF–DC converter adopts a dual-band impedance-matching circuit for RF energy harvesting in multiple frequency bands and an adaptive configuration that operates in an optimal configuration for high efficiency by changing the operation mode according to the RF input power. Since the optimum peak PCE can be obtained according to the RF input power, the PCE can be increased over a wide RF input power range of multiple bands. When dual-band RF input powers of 0.9 GHz and 2.4 GHz were applied, a peak PCE of 67.1% at an input power of −12 dBm and a peak PCE of 62.9% at an input power of −19 dBm were achieved, respectively. The input sensitivity to obtain an output voltage of 1 V was −17 dBm, and the RF input power range with a PCE greater than 20% was 21 dB. The proposed design achieved the highest peak PCE and the widest RF input power range compared with previously reported CMOS multi-band rectifiers. 

## Figures and Tables

**Table 1:** Performance results at different temperatures.

Temperature	0 °C	27 °C	70 °C
*S*_11_	−14.4 dB @ 0.9 GHz	−15.8 dB @ 0.9 GHz	−10.3 dB @ 0.9 GHz
	−15.8 dB @ 2.4 GHz	−17.8 dB @ 2.4 GHz	−14.7 dB @ 2.4 GHz
Output voltage	0.905 V @ −19 dBm	0.890 V @ −19 dBm	0.856 V @ −19 dBm
	2.082 V @ −12 dBm	2.057 V @ −12 dBm	2.003 V @ −12 dBm
Peak PCE	68.7%	67.1%	63.6%

**Table 2:** Performance summary and comparison.

Reference	[5]	[11]	[12]	[13]	This Work
CMOS technology	65 nm	130 nm	180 nm	180 nm	180 nm
Frequency	0.9 GHz	0.9/2.0 GHz	0.93/2.63/ 0.93 + 2.63 GHz	0.9/2.45 GHz	0.9/2.4/ 0.9 + 2.4 GHz
Peak PCE (@ RF input power)	36.5% (@ −10 dBm)	9.1% (@ −19 dBm)	23.3% (@ −1 dBm)	47% (@ 1 dBm)	67.1% (@ −12 dBm)
Input power range for PCE > 20%	11 dB	N.A.	N.A.	19 dB	21 dB
Sensitivity (for *V_DC_* = 1 V)	−17.7 dBm	−19 dBm	−15.4 dBm	N.A.	−17 dBm
*R_L_*	147 kΩ	1 MΩ, 1.5 MΩ	500 kΩ	5 kΩ	100 kΩ
